# Ultraviolet B (UVB) Irradiation-Induced Apoptosis in Various Cell Lineages *in Vitro*

**DOI:** 10.3390/ijms14010532

**Published:** 2012-12-27

**Authors:** Sara Salucci, Sabrina Burattini, Michela Battistelli, Valentina Baldassarri, Maria Cristina Maltarello, Elisabetta Falcieri

**Affiliations:** 1 DiSTeVA, University of Urbino “Carlo Bo”, Urbino 61029, Italy; E-Mails: sara.salucci@uniurb.it (S.S.); sabrina.burattini@uniurb.it (S.B.); michela.battistelli@uniurb.it (M.B.); valentina.baldassarri@uniurb.it (V.B.); 2 Laboratory of Musculoskeletal Cell Biology, Rizzoli Orthopaedic Institute, Bologna 40136, Italy; E-Mail: mariacristina.maltarello@ior.it; 3 IGM, CNR, Rizzoli Orthopaedic Institute, Bologna 40136, Italy

**Keywords:** UVB, apoptosis, HL-60, Molt-4, U937, chondrocytes, skeletal muscle cells

## Abstract

Ultraviolet B (UVB) radiation acts as a strong apoptotic trigger in many cell types, in tumor and normal cells. Several studies have demonstrated that UVB-induced cell death occurs through the generation of reactive oxygen species. The consequent oxidative stress includes the impairment of cellular antioxidants, the induction of DNA damage and the occurrence of apoptosis. In this review, we investigated UVB apoptotic action in various cell models by using ultrastructural, molecular and cytofluorimetric techniques. Myeloid leukemia HL-60, T-lymphoblastoid Molt-4 and myelomonocytic U937 human cells, generally affected by apoptotic stimuli, were studied. Human chondrocytes and C2C12 skeletal muscle cells, known to be more resistant to damage, were also considered. All of them, when exposed to UVB radiation, revealed a number of characteristic apoptotic markers. Membrane blebbing, cytoplasm shrinkage and chromatin condensation were detected by means of electron microscopy. DNA cleavage, investigated by using agarose gel electrophoresis and TUNEL reaction, was observed in suspended cells. Differently, in chondrocytes and in skeletal muscle cells, oligonucleosomic DNA fragmentation did not appear, even if a certain TUNEL positivity was detected. These findings demonstrate that UVB radiation appears to be an ideal tool to study the apoptotic behavior.

## 1. Introduction

UVB radiation-induced apoptosis has been extensively studied in human keratinocytes, which represent the major cellular target for solar UVB radiation [1]. Skin cells undergo apoptosis because of irreversible DNA damage [2], and it can prevent the accumulation of abnormal cells, which could lead to cutaneous malignancies [3].

The molecular pathways leading to UVB radiation-induced apoptosis include the formation of cyclobutane pyrimidine dimers (CPDs) and photoproducts [4,5], the activation of death receptors, including CD95 (Fas/APO-1) [4,6], and the formation of reactive oxygen species (ROS) [7].

Recently, several studies have demonstrated that many harmful effects of short-wavelength UVB rays (290–320 nm) occur through the generation of ROS [8–10]. Oxidative stress response of cells and tissues includes the induction of oxidative damage to various cellular components (e.g., membrane lipids, proteins and DNA) and the occurrence of inflammation, immunosuppression and apoptosis [11–15]. After UVB exposure, DNA double-strand breaks occur; they are particularly toxic and may not be correctly repaired. This could lead to chromosomal translocations and to the formation of highly unstable dicentric chromosomes or acentric chromosomal fragments [16], which are correlated to the induction of apoptosis [17].

The purpose of this review is to report and discuss the UVB radiation role in apoptotic cell death, induced *in vitro* in different cell lines.

In fact, UVB radiation is a known inducer of apoptosis in cultured cells [18–21]. It can trigger both the extrinsic and the intrinsic apoptotic pathways, but it remains unclear how these pathways are interrelated [22]. Recent studies demonstrated that UVB-induced cell death mostly occurs through the intrinsic apoptotic pathway [23,24], even if the presence of caspase-independent mechanisms cannot be excluded. Anyhow, a mitochondrial involvement in UVB-induced apoptosis is certain. In fact, it is well known that UVB radiation alters the structure of the outer mitochondrial membrane, causing its permeabilization and the cytochrome c release [24–26].

Cell exposure to UVB is one of the best experimental systems to study apoptosis in response to DNA damage [27,28]. Morphological observations showed that low doses of UV induced apoptosis [27], whereas higher doses triggered both apoptosis and necrosis [29]. UVB, which is an oxidant and pro-apoptotic agent widely demonstrated in keratinocytes, melanocytes and epidermal cells [30–32], appeared also useful to study apoptotic behavior in other cell cultures *in vitro.*

In the early years of our research, the UVB apoptotic effect was analyzed in different models: myeloid leukemia HL-60, T-lymphoblastoid Molt-4 and myelomonocytic U937 human tumor cell lines that grow in suspension. They have a high proliferative rate and, for this reason, they are usually more sensitive to cell death stimuli. As described in the literature, UVB radiation induces apoptosis rapidly and essentially in the entire cell population (about 90%). This aspect has been discussed in several reports in which apoptosis of human myeloblastic cells, induced by UV radiation, has been investigated [18,33].

Several studies, in the last decade, showed that apoptosis, necessary for maintaining tissue homeostasis, plays an active role not only in carcinogenesis [7,34]. It contributes to articular cartilage damage in osteoarthritis, and it is correlated to a number of cartilage disorders [35]. It is also necessary for the regulation of skeletal muscle homeostasis, and it has been described in several myopathies, as well as after denervation or disuse [36,37].

Thus, the UVB-induced DNA damage was recently investigated by our group in human chondrocytes and in C2C12 murine skeletal muscle cells, both known to be somehow apoptosis-resistant. Differently from suspended cell models, routinely used to study apoptosis, primary cell lines (chondrocytes) and murine skeletal myoblasts originally derived from satellite stem cells appeared as complex cell systems in which several molecules and genes are involved in promoting cell survival or driving the apoptotic process. For these reasons, they can be considered more resistant to apoptotic induction if compared to leukemic cells.

In this review, to obtain a massive apoptosis, cells were exposed to UVB (dose 72 J/cm^2^, wavelength 312 nm, Transilluminator 2000, Bio-Rad Laboratories, Hercules, CA, USA) for 30 min at room temperature, at a distance of 1 cm, and then post-incubated for 4 h at 37 °C in humidified air with 5% CO_2_. Different UVB exposure times were considered for chondrocytes and C2C12 cells, but after 1 h of treatment, a massive necrotic cell death appeared. Thus, 30 min of UVB exposure has been chosen for our experiments with all cell types [18,25].

This review demonstrates that UVB radiation induces apoptosis in various cell lines using different approaches: Scanning (SEM) and Transmission (TEM) Electron Microscopy used to evidence morphological features, such as surface blebbing, cytoplasm shrinkage and chromatin condensation [38–41]; agarose gel electrophoresis and TUNEL reaction, the latter both at fluorescence and electron microscopy, used to investigate DNA [42–44]; and flow cytometry used to analyze cell cycle patterns and mitochondrial activity [45,46].

### 1.1. Human Hemopoietic Cells

HL-60 (human myeloid leukemia cells), Molt-4 (T-lymphoblastoid cells) and U937 (myelomonocytic human leukemia cells) are round mononuclear cells, with microvilli. It is known that HL-60 and other human cell lines were found to rapidly undergo apoptosis after short periods of UV radiation, whereas prolonged exposure induced necrotic cell death [47].

**Figure 1:**
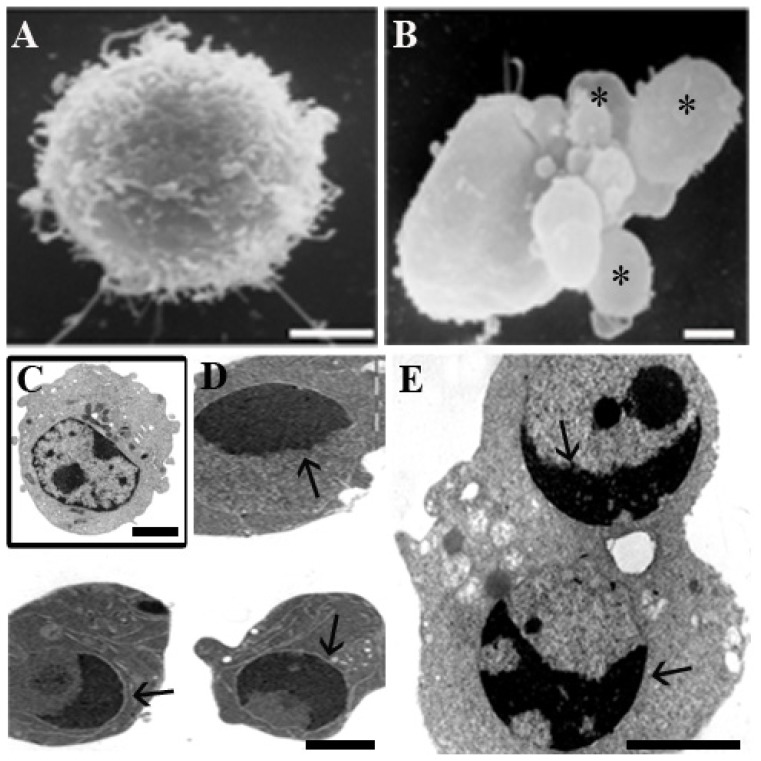
Control (**A**) and Ultraviolet B (UVB)-treated (**B**) HL-60 cells at Scanning Electron Microscopy (SEM). Surface blebbing (**B**, asterisks), absent in the control (**A**), appears. Control (**C**) *vs*. treated U937 (**D**) cells and treated Molt-4 (**E**) cells at Transmission Electron Microscopy (TEM). Condensed chromatin, organized in cup-shaped masses (arrows) under the nuclear envelope, can be observed in both cases (**D**,**E**). Scale bars: **A**–**C**, 2 μm; D,E, 1 μm.

Thus, cells were exposed to 30 min UVB, post-incubated for 4 h in growing media and then processed for ultrastructural, molecular and cytofluorimetric investigations. After UVB treatment, all cell types showed apoptotic patterns (Figure 1). Ultrastructural observations evidenced surface blebbing (Figure 1B), cytoplasm shrinkage and chromatin condensation (Figure 1D,E) [43,46]. Control and UVB-treated specimens were processed for SEM and TEM, as previously reported [39].

**Figure 2:**
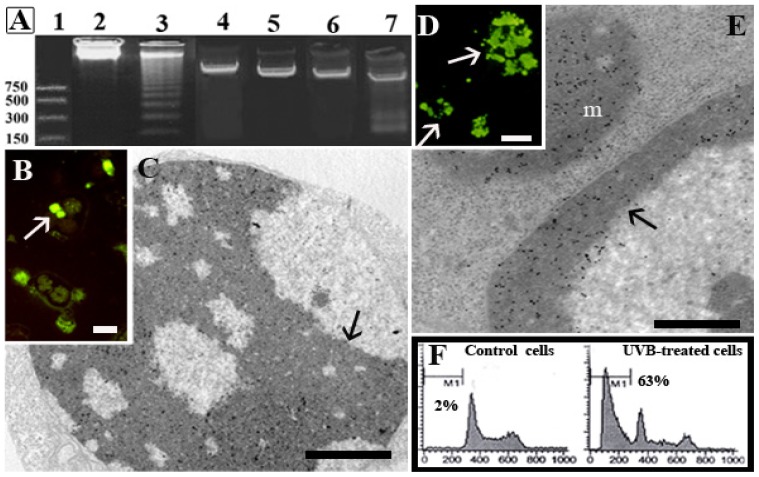
In HL-60 (lane 2), Molt-4 (lane 4) and U937 (lane 6) control cells, as well as in UVB-treated Molt-4 (lane 5), DNA cleavage is absent (**A**). In treated HL-60 (lane 3) and U937 (lane 7) cells, the ladder, due to oligonucleosomic fragmentation, appeared. Terminal deoxynucleotidyl transferase-mediated dUTP nick-end labeling reaction at fluorescence microscope (TUNEL/FM) (**B**,**D**) and TUNEL/TEM (**C**,**E**) of Molt-4 (**B**,**C**) and U937 (**D**,**E**) cells. Fluorescent micronuclei (arrows) are evident in both cases (**B**,**D**). Colloidal gold particles can be detected in dense chromatin (**C**,**E**, arrows) and micronuclei (m) appear labeled (**E**). U937 cell DNA content, evaluated by flow cytometry is shown in (**F**). A sub-G1 peak (63%) is revealed in treated cells, whereas it is absent in the control ones. Scale bars: (**B**,**D**) 20 μm; (**C**) 0.5 μm; (**E**) 0.1 μm.

Renò *et al.* 1998 [48] studied the plasma membrane behavior in HL-60 and Molt-4 cells after UVB exposure, to investigate its involvement in apoptosis. The results showed that during the early stages of apoptosis, a membrane lipid rearrangement occurs and involves phosphatidylserine translocation from the inner to the outer leaflet, independently from nuclear activity. Moreover, in Figure 2, DNA behavior has been also investigated showing that in HL60, a widely studied leukemia cell line, the oligonucleosomic DNA cleavage occurred (Figure 2A, lane 3). On the other hand, in Molt-4, oligonucleosomic DNA fragmentation was not observed (Figure 2A, lane 5), even in the presence of typical apoptotic features: chromatin condensation, cell shrinkage with preservation of the plasma membrane structure, nuclear splitting and micronuclei formation. Molt-4 cell response to UVB was investigated not only at the standard post-incubation time (*i.e.*, 4 h), but also at 6, 8, 10 and 24 h and DNA cleavage never occurred [44]. The lack of endonuclease activation can be postulated in this apoptotic model, despite the existence of a metabolic pathway responsible for chromatin rearrangement and other apoptotic patterns [38]. In addition, after conventional agarose gel electrophoresis, a typical DNA ladder appeared in U937 cells, too (Figure 2A, lane 7) [44], as also described in another work [49].

Cells were processed for TUNEL reaction at fluorescence microscope (TUNEL/FM). Moreover, to better highlight DNA fragmentation, the same technique was adapted for electron microscopy (TUNEL/TEM) [50,51]. In this way, a precise localization of DNA break points within the different chromatin domains was obtained (Figure 2). In UVB-treated Molt-4 cells, TUNEL/FM showed several positive cells (Figure 2B), also observed by TUNEL/TEM. The latter revealed a number of nuclear gold particles in the dense chromatin (Figure 2C). A peculiar chromatin behavior, reported to as “moth-eaten” [45], was observed in this cell lineage exposed to UVB, where small negative areas of diffuse chromatin scattered throughout the positive dense chromatin.

Numerous U937 cells with homogeneously fluorescent micronuclei (Figure 2D) appeared at TUNEL/FM. An intense gold labeling (TUNEL/TEM) was observed in dense apoptotic chromatin and micronuclei (Figure 2E) [52].

Moreover, Luchetti *et al.* 2006 [46] studied melatonin antiapoptic activity in UVB-treated U937 cells, analyzing the cell cycle profile by means of flow cytometry. A conspicuous hypodiploid peak appeared after UVB treatment (Figure 2F), revealing an apoptotic cell population with DNA cleavage also evidenced by Liu *et al.* 2005 [53] in the study on oridonin role in enhancing phagocytosis of UV-irradiated apoptotic U937 cells. Moreover, in this cell line, mitochondrial activity was investigated using mitochondrial fluorescent probes, such as Mito Tracker and JC-1, that revealed an alteration of mitochondrial membrane potential. This event has been evidenced using the cardiolipin-sensitive probe 10-nonyl acridine orange (NAO), to monitor changes in mitochondrial lipids. A decrease in cardiolipin content, induced by ROS increase, occurred in concomitance with mitochondrial permeability alteration and, successively, with the release of cytochrome c into the cytosol [54].

### 1.2. Chondrocytes

After local Ethics Committee approval, fragments of articular cartilage were obtained from 16 patients (mean age 67 years, range 41–81 years) who were undergoing knee replacement. The tissue was finely minced and subject to enzymatic digestion; primary chondrocytes were cultured in micromass [35], which represent a convenient model to study chondrocyte biology [55] and, in particular, their death, in the context of a tridimensional culture model.

**Figure 3:**
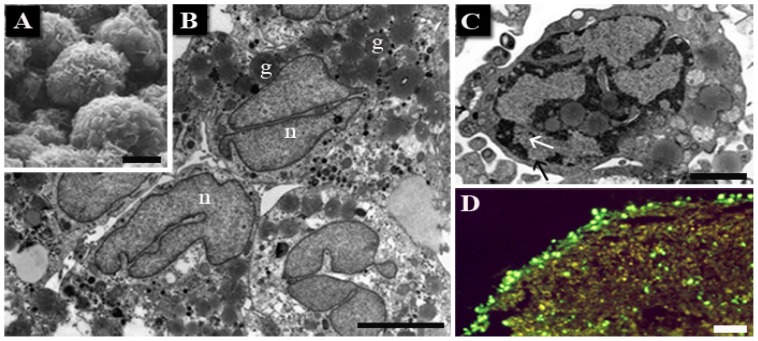
Untreated chondrocytes at SEM (**A**) and TEM (**B**). Condensed chromatin can be observed after UVB treatment (**C**), where pore translocation appears at diffuse chromatin level (arrows). Nuclear TUNEL-positivity is shown at fluorescence microscope (**D**). n: nuclei; g: lipid granules. Scale bars: (**A**–**C**) 5 μm; (**D**) 40 μm.

Chondrocyte morphology (Figure 3) in control condition appears very similar to that of human articular cartilage. Cells are round or slightly elongated with a plurilobated nucleus and dispersed chromatin. Large amount of glycogen masses and lipid granules can be observed scattered throughout the cytoplasm. Proteoglycans and collagen fibers are present in the intercellular space, indicating a good extra-cellular matrix production (Figure 3A,B).

After UVB treatment, chromatin condensation appears, even if dense cup-shaped masses, comparable to those of more classic apoptotic models, could not be found. The evidenced nuclear features, when analyzed in detail, suggest apoptosis [56].

Gel electrophoresis did not show oligonucleosomic DNA cleavage (data not shown). Nevertheless, after UVB exposure, TUNEL evidenced the presence of positive nuclei, in particular in chondrocytes at the micromass periphery (Figure 3D).

### 1.3. Skeletal Muscle Cells

Murine C2C12 myoblasts have been widely used as a model to study apoptosis in developing muscle, because extensive cell death occurs during myogenic differentiation [57].

**Figure 4:**
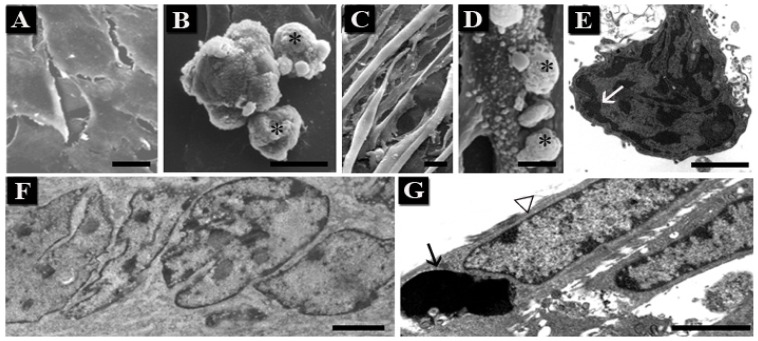
Surface blebs (asterisks), absent in control myoblasts (**A**) and myotubes (**C**), appear after UVB treatment (**B** and **D**) at SEM. Myoblast chromatin condensation (arrow) can be also revealed (**E**). TEM of control (**F**) and treated myotubes (**G**) shows nuclear peculiarities. In the latter normal (arrowhead) and apoptotic (arrow) nuclei coexist (**G**). Scale bars: (**A**–**D**) 10 μm; (**E**,**G**) 2 μm; (**F**) 2.5 μm.

C2C12 is an adherent cell line of murine myoblasts that can be induced to differentiate *in vitro* into multinucleated myotubes, which progressively become muscle fibers [58]. These cells are considered apoptosis-resistant, in particular myotubes [59], but when exposed to UVB, undifferentiated and differentiated C2C12 showed a certain number of apoptotic features (Figure 4). Most cells began to detach from the substrate and membrane blebbing could be observed at SEM (Figure 4B,D).

Apoptotic nuclei showed condensed chromatin, which marginated beneath the nuclear membrane, occasionally forming micronuclei, even if the classical cup-shaped masses were not visible (Figure 4E). In addition, the simultaneous presence of normal and apoptotic nuclei inside the same myotube (Figure 4G) has been observed at TEM, suggesting a compartmentalization in nuclear domains. In the report by D’Emilio *et al.* [25] we hypothesized, indeed, the presence of territorial areas, which maintain along the syncytium, a certain specificity and the capability to differently respond to external stimuli.

**Figure 5:**
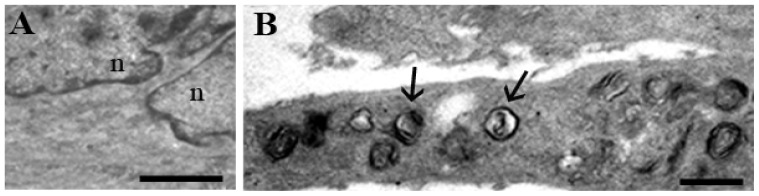
Autophagy occurs in UVB-radiation response of differentiated C2C12 cells. Autophagic vacuoles (arrows), absent in C2C12 control myotubes (**A**), appeared in UVB-treated myotubes (**B**). n: nuclei. Scale bars: (**A**) 2.5 μm; (**B**) 1 μm.

Furthermore, after UVB radiation of C2C12 muscle cells, autophagy appeared (Figure 5). This process, recognizable by the presence of cytoplasmic vacuoles filled with membranes, organelles and mitochondria remnants, is clearly evident in treated myotubes, while absent in control condition.

**Figure 6:**
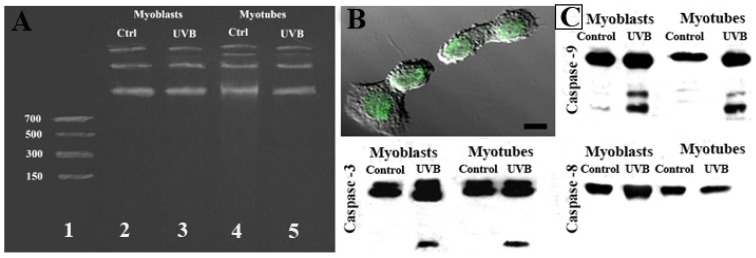
DNA cleavage (**A**) is absent in control myoblasts (lane 2) and myotubes (lane 4) and in UVB-treated myoblasts (lane 3) and myotubes (lane 5). Nevertheless, TUNEL positive nuclei were observed in treated myoblasts at confocal microscopy (**B**). Caspase-9 and -3 activation and caspase-8 absence were demonstrated by Western blotting, both in myoblasts and myotubes (**C**). Scale bar: (**B**) 20 μm.

In Figure 6, agarose gel electrophoresis did not show oligonucleosomic DNA cleavage in myoblasts nor in myotubes (Figure 6A), but the TUNEL technique revealed a diffuse positivity (Figure 6B) and confirmed the presence of positive and negative nuclei inside the same myotube (data not shown). Moreover, molecular analyses evidenced, in both myoblasts and myotubes, the activation of caspase-9 and -3, but not caspase-8 (Figure 6C). D’Emilio *et al.* 2010 [25], demonstrated that caspase-9 and -3 inhibitors only partially reduced the apoptotic rate, suggesting that apoptosis occurred by following both caspase-dependent and -independent pathways.

## 2. Conclusions

In this review, the apoptotic behavior of different cell lineages after UVB exposure has been investigated by means of several techniques, evidencing that UVB radiation plays a central role in the induction of apoptotic cell death. UVB is a powerful apoptotic stimulus in hemopoietic tumor cells. In these models, classical morphological apoptotic features were indeed observed, together with oligonucleosomic DNA cleavage evidenced after electrophoresis, TUNEL-FM and -TEM and cell cycle analysis. Cytofluorimetric analyses, used to test mitochondrial activity, showed that UVB induced cardiolipin decrease with alteration of mitochondrial membrane permeabilization and, as a consequence, cytochrome c release [54]. In suspended cells, UVB induced apoptosis mostly follows the mitochondrial pathway. Luchetti *et al.* 2009 [18] evidenced that melatonin was able to downregulate superoxide anion production, mitochondrial damage and caspase-dependent apoptosis in U937 cells exposed to UVB. In keratinocytes, UV-induced apoptosis is a complex event and involves different pathways. Apoptosis can be triggered by direct DNA damage, ROS production and death receptor activation and mitochondrial damage [12,27,60–62]. Several studies [26,63] discussed the molecular mechanisms of UV-induced apoptosis in keratinocytes, demonstrating that mitochondrial changes and cytochrome c release are involved and can be considered the predominant mechanisms in UV-mediated apoptosis.

UVB application to other cell models has been considered; chondrocytes and skeletal muscle cells, even if known to be resistant to apoptosis, were studied. Intriguingly, chondrocytes appeared sensitive to UVB treatment, at ultrastructural and molecular analyses, showing chromatin condensation, pore clustering and DNA fragmentation only revealed by TUNEL reaction, whereas DNA fragmentation was absent.

Moreover, as also reported by D’Emilio *et al.* 2010 [25], skeletal muscle cells, as undifferentiated myoblasts and in the form of highly differentiate myotubes, appeared sensitive to UVB-induced apoptosis.

Chromatin changes, with progressive compaction and formation of micronuclei, were identified after ultrastructural observations. In this cell line, oligonucleosomic DNA cleavage does not seem to occur [15], but double-strand DNA breaks are shown by TUNEL reaction. The presence of normal and apoptotic nuclei inside the same syncytium seems to indicate that each myonucleus regulates a certain fiber volume, suggesting a cytoplasmic compartmentalization in nuclear domains. Thus, individual myonuclear apoptosis, as well as complete cell death, can occur, as previously described by other authors [64,65]. The partial inhibition of apoptosis by caspase inhibitors (both -9 and -3) indicates that UVB radiation is able to trigger apoptosis through the activation of the mitochondrial pathway, as occurs in epidermal cells [32]. Moreover, as reported by Sitailo *et al.* 2002 [63], in keratinocytes, UV radiation triggers activation of caspase-3, -9 and -8. UVB radiation activates primarily the mitochondrial or intrinsic apoptotic pathway, resulting in activation of procaspase-9, whereas activation of procaspase-8 via death receptors is a relatively minor pathway. As a consequence, UVB is able to induce apoptosis through the mitochondrial pathway in all cell systems considered in this review, as well as in epidermal cells.

Furthermore, molecular analyses demonstrated that DNA cleavage on agarose gel electrophoresis appeared only in U937 and HL-60 cell lines. Molt-4 cells exposed to UVB showed the lack of DNA fragmentation even if morphological apoptotic features appeared in the presence of TUNEL reaction positivity, detected both by fluorescence and electron microscopy. The lack of DNA cleavage can be linked to endonuclease absence [66] or, as suggested by Yanagisawa-Shiota *et al.* 1995 [67], endonuclease activity might be differently regulated in myelogenous (U937, HL-60) and non-myelogenous leukemic cell lines (Molt-4).

In our experiments, oligonucleosomic DNA fragmentation did not appear after UVB exposure in chondrocytes and skeletal muscle cells. The DNase responsible for the apoptotic DNA laddering and the precise molecular mechanisms are poorly understood in the case of these cell lines. Shiokawa *et al.* 2002 [57] demonstrated that an olinucleosomic DNA fragmentation occurred in myoblasts only when apoptosis appeared during induction of differentiation.

Moreover, in muscle cells, autophagy was observed after UVB exposure. Recent papers, evaluating the induction of LC3 lipidation and the increase of beclin-1 expression, demonstrated that UVB radiation induces autophagy in epidermal cells [68], where it appeared to be a protective response to damage. Inhibition of autophagy exacerbated UVB-induced cell death, whereas its stimulation provided protection [69,70]. It is known that autophagy is an important catabolic program fundamental for tissue homeostasis; in fact, it promotes physiological protein turnover and removes damaged proteins and organelles [69]. However, the role of autophagy as a mechanism of cell survival or death is still controversial, even if some evidences demonstrated its protective action against cell death after UV radiation [71].

In C2C12 control cells, as demonstrated by our images, autophagic vacuoles were not observed. After UVB-induced cell death, both myoblasts and myotubes evidenced a certain number of autophagic vacuoles, which appeared to be more diffuse in cells that do not show apoptotic changes.

Finally, these data demonstrated that UVB radiation is ideal to trigger apoptosis in different cell models, where they mainly act through the mitochondrial pathway. Thus, in all cell types considered in this review, as well as in skin cells, which are the major target of solar radiations, UVB seems to induce apoptosis through the same pathway. Unexpectedly, in skeletal muscle cells, as well as in keratynocytes, as recently evidenced by other works, UVB radiation induced autophagy; further studies are required to investigate its role through molecular and biochemical approaches.
